# Pharmacogenomics in the UK National Health Service: Progress towards implementation

**DOI:** 10.1002/bcp.70109

**Published:** 2025-06-03

**Authors:** John H. McDermott, Maria Tsakiroglou, William G. Newman, Munir Pirmohamed

**Affiliations:** ^1^ Manchester Centre for Genomic Medicine, St Mary's Hospital Manchester University NHS Foundation Trust Manchester UK; ^2^ Division of Evolution and Genomic Sciences, School of Biological Sciences University of Manchester Manchester UK; ^3^ Department of Pharmacology and Therapeutics, Wolfson Centre for Personalised Medicine University of Liverpool Liverpool UK; ^4^ Liverpool University Hospital Foundation NHS Trust Liverpool UK

**Keywords:** clinical pharmacology, genetics and pharmacogenetics, medication safety, pharmacogenomics

## Abstract

Over the past decade there has been considerable and growing enthusiasm about the promise of using genomics to inform healthcare. In particular, using genetic data to inform prescribing practice has emerged as a compelling policy priority for health systems around the world, not least in the NHS. Various initiatives and strategies have been developed to explore the value of pharmacogenomics in the UK National Health Service (NHS) and identify strategies for implementation. The NHS England Network of Excellence for Pharmacogenomics and Medicines Optimisation (PGx‐NoE) was launched in 2024 and held two stakeholder meetings over the year in collaboration with the UK Pharmacogenetics and Stratified Medicine Network and the British Pharmacological Society (BPS). This article describes the outputs of those meetings, which are discussed in the context of previously identified challenges and opportunities. Rather than simply identify further barriers or facilitators, outputs are contextualized around tangible recommendations and real‐world implementation exercises. These are grouped into three key areas: genetics, data and service. The work of partners across the UK are highlighted, including development of the NHS England Genomic Test Directory, the proof‐of‐principle informatic patterns demonstrated by the PROGRESS study, and the launch of the Centre for Excellence in Regulatory Science and Innovation (CERSI) in Pharmacogenomics, which will create UK‐specific guidance and clarify complex regulatory pathways. Many of the well‐defined barriers to the implementation of pharmacogenomics have been addressed in recent years, and this work highlights how the UK has the opportunity to emerge as a global leader in genomics‐informed healthcare.

## 1 INTRODUCTION

The NHS is an umbrella term for the publicly funded healthcare systems of the United Kingdom (UK), with Scotland, Wales and Northern Ireland each having devolved responsibilities for the planning and provision of healthcare. Over 90% of all healthcare in the UK is funded by the NHS, and in 2023/24 its budget was £188.5 billion.^1^, ^2^ The funding pressures facing the NHS are well documented, and in an effort to reform the health service and ensure its financial sustainability, three major strategic shifts were announced by the Labour government in 2024.^3^ One of these is to shift the model of healthcare from sickness to prevention, keeping people healthy for longer and reducing the burden on the NHS. Ensuring patients are taking the right medicines to treat and prevent disease is a major component of this, and there have been growing efforts for several years to explore how genomics might contribute towards this effort.

In 2019, a workshop was convened in London to discuss how genomic data might be integrated into routine healthcare to improve prescribing practice, a concept known as pharmacogenomics. This brought together key stakeholders from across the health system with representation from the UK Pharmacogenetics and Stratified Medicine Network, NHS England and Genomics England. A subsequent perspective from Turner et al. summarized the meeting's findings (Table 1) and highlighted the major issues and challenges preventing the implementation of pharmacogenomics in the NHS and started to define potential solutions.^4^

**TABLE 1:** Defining the issues, challenges and progress towards the implementation of pharmacogenomics in the NHS.

	Turner et al. (2020)	Personalised Prescribing report (2022)	NoE meeting (2024)
	Issues	Key recommendations	Progress assessment and next steps
***Genetics***	No consensus regarding the technical approach to genetic testing	(1) Transition towards panel‐based pharmacogenomic testing rather than single‐gene approaches.(2) Explore the extraction of pharmacogenomic data from sequence data performed for other indications.	Several pharmacogenetic tests are now available on the NHS Test Directory. Panel‐based testing trialled within the PROGRESS study.
	Uncertainty regarding which gene–drug pair associations to include	(3) Centralized selection of key gene–drug pairs for initial national rollout. (4) Specify clinical indication(s) for testing each gene–drug pair in keeping with guidelines and prescribing information from regulatory agencies such as the MHRA.	Test directory acts as centralized resource for testing. CERSI established to create UK consensus guidance for pharmacogenetic‐guided prescribing.
***Data***	Limited capacity for testing	(5) Determine anticipated testing volumes to not overload the nascent system. (6) Plan for incremental and iterative service expansion.	Testing volumes are currently managed by eligibility criteria. Point of care technologies have been deployed in several settings where a rapid result is required. To scale, dedicated pharmacogenetic laboratory capacity is needed.
	Lengthy turnaround times	(7) Ensure the test turnaround time is congruent with the chosen patient pathways. (8) Use PoC testing when pharmacogenomic data is required rapidly.	
	Complexity of interpretation	(9) Develop clinical pharmacogenomics guidance tailored to the NHS.	Presented data to clinical stakeholders as actionable guidance, as per PROGRESS.
	Lack of decision support systems	(10) Build interconnected systems that enable access to pharmacogenomic data across the healthcare system.	A pilot platform to deliver guidance via CDS directly within EHRs has been developed by the PGx‐NoE.
***Service***	Conflicting evidence for clinical effectiveness	(11) Improvement of the recording of treatment to improve measures of outcome and impact.	There should be ongoing monitoring of implementation programmes. Routinely collected healthcare data should be used to capture these outcomes.
	Uncertainty regarding cost‐effectiveness	(12) Conduct collaborative, inclusive and multidisciplinary research to transition towards a learning healthcare system that provides equitable, clinically and cost‐effective accessible and acceptable care.	NICE have undertaken cost‐effectiveness analysis for a number of single gene–drug pairs. There is a need to better assess the cost effectiveness of panel‐based testing.
	Lack of public and healthcare professional awareness	(13) Patient representatives should be involved throughout the implementation and service expansion phases. (14) Service delivery must be receptive and responsive to patient feedback. (15) Just‐in‐time resources used at or near the point of prescribing.	Just‐in‐time resources have been developed via the Genomics Education Programme and should be integrated into CDS.

Abbreviations: CDS: clinical decision support; CERSI: Centres of Excellence in Regulatory Science and Innovation; EHR: electronic health records; MHRA: Medicines and Healthcare products Regulatory Agency; NICE: National Institute for Health and Care Excellence; NoE: Network of Excellence; PGx‐NoE: Network of Excellence for Pharmacogenomics and Medicines Optimisation; PoC: point‐of‐care; PROGRESS: Gauging Response to Service.

*Note*: This table was developed by extracting the key issues defined by Turner et al. and mapping these against the recommendations outlined in the Personalised Prescribing report. Areas of progress were contemporaneously recorded during the 2024 NoE meeting and then mapped against the appropriate recommendation. Mapping was agreed between all authors.

Since the publication of this perspective in 2020, a number of policy and healthcare initiatives have combined in the NHS to create an organizational framework which is now able to advance the implementation of pharmacogenomics.^4^, ^5^ In England, the Accelerating Genomic Medicine strategy was published in 2022, beginning with the core vision to ensure that “the power of genomics in predicting, preventing and diagnosing disease, and targeting treatment is accessible to all as part of routine care in the NHS”.^6^ The strategy outlined a series of key priorities, the majority of which were related to rare disease or cancer. However, the “emerging field” of “pharmacogenetics” received particular attention.

More recently, the Royal College of Physicians (RCP) of London, and the British Pharmacological Society (BPS), jointly published a report, “Personalised Prescribing”, which called for the implementation of pharmacogenetic testing in the NHS and was informed by the 2020 perspective by Turner et al., providing some more tangible recommendations to address the implementation gap.^5^ This report recommended that the NHS should transition towards a pre‐emptive panel‐based genotyping approach and data should be made available to primary, secondary and tertiary care settings, now complementing the recently announced “strategic shift” to move from sickness to prevention. Furthermore, the authors recommended that the clinical effectiveness of the pharmacogenetics implementation service should be subject to continuous and iterative evaluation with the aim of ensuring that the service has maximum utility and efficiency. Funding for pharmacogenetic testing, the report said, should be made available from central, rather than local, funds to avoid geographical inequalities.

A major challenge in delivering on these broad ambitions is that defining the relevant people, systems, needs and risks across the NHS for a potentially ubiquitous intervention like pharmacogenetics is highly complex. Recognizing this, in 2023 NHS England established the Network of Excellence for Pharmacogenomics and Medicines Optimisation (PGx‐NoE). This structure aims to bring together multidisciplinary expertise from around the country to develop a single strategy for the implementation of pharmacogenomics in England, with input from representatives of each of the seven Genomic Medicine Service Alliance (GMSA) regions. This structure is well placed to address the issues outlined by Turner et al. in 2020, leveraging the recommendations set out in the Personalised Prescribing report.

Five years on from the workshop in London, which first systematically characterized the challenges preventing the implementation of pharmacogenomics in the NHS, the UK Pharmacogenetics and Stratified Medicine Network supported by the BPS and the PGx‐NoE hosted a joint meeting to assess progress towards implementation.^7^ Stakeholders from the NHS, academia and industry attended to discuss national and regional implementation programmes, focusing on areas of best practice. Recommendations in the Personalised Prescribing report can be mapped to each of the nine key issues highlighted during the original workshop as barriers to the implementation of pharmacogenomics in the NHS, providing a framework against which progress can be assessed in this report (Table 1).

## 2 THE GENETICS

### 2.1 Genetic testing approaches

Using pharmacogenetics to guide prescribing practice requires the results of a genetic test to be embedded within a model of service delivery which clinical and public stakeholders can access and ultimately want to use. In other words, when considering developing a pharmacogenetic service, one must consider (a) the genetic testing approach (the test), (b) how that test is ordered and the results then used (the service), and (c) the stakeholder acceptability of (a) and (b) (Table 2).

**TABLE 2:** Testing approaches and models of service delivery.

	Single gene testing	Panel‐based testing
**Reactive**	Single gene testing with no reuse of data for future prescribing. *Predominant approach in the NHS at present –* e.g.*, DPYD & Fluoropyrimidines*.	Panel testing with no reuse of data for future prescribing. *Not used in the NHS in the context of pharmacogenomics. Represents a poor use of additional genetic data included on the panel*.
**Reactive with planned reuse**	Single gene testing in the context of a prescription, with data then reused to inform future prescribing. *Not used in the NHS. Theoretically, an individual might have CYP2C19 testing to guide clopidogrel prescription, then the same information be used to inform the prescription of a proton pump inhibitor*.	Panel testing in the context of a prescription, with the data then reused to inform future prescribing. *Not used in the NHS. A model increasingly applied by pharmacogenetic programmes globally, and recently trialled by the PREPARE study*.^8^, ^9^
**Pre‐emptive**	Single gene testing with data stored in medical records to inform future prescribing practice. *RNR1 (m.1555A>G) testing in a child diagnosed with cystic fibrosis, with result stored in records to inform future antibiotic prescription*.^10^, ^11^ *Otherwise, not used in the NHS*.	Panel testing not performed within the context of a specific prescription, with the data then reused to inform future prescribing. *Not used in the NHS. Fully pre‐emptive pharmacogenetic testing has been trialled in a small number of biobank studies*.

*Note*: Theoretical pharmacogenetic implementation strategies, considering the testing approach and model of service delivery. PREPARE: Pre‐emptive Pharmacogenomic Testing for Preventing Adverse Drug Reactions.

Although an important consideration, the question of the test in this context does not relate to the technology itself (i.e. Sanger sequencing *vs*. next generation sequencing) but refers to the extent of genetic information produced. Pharmacogenetic testing can either be “single gene”, where variants in just one gene are tested and reported, or “panel‐based”, where variants across several genes are analysed at the same time.^4^, ^12^ Single gene pharmacogenetic testing is currently performed in the NHS (Table 3). However, the Pre‐emptive Pharmacogenomic Testing for Preventing Adverse Drug Reactions (PREPARE) study within the European Ubiquitous Pharmacogenomics (U‐PGx) project provided some evidence that use of pharmacogenomic panel‐based testing (12 genes and 44 variants) to guide prescribing can reduce adverse drug reactions by up to 30%.^8^

**TABLE 3:** Pharmacogenetic testing available in England.

Medicine	Gene(s)	Variant/haplotype testing available in the NHS	Notes
Fluoropyrimidines	*DPYD*	c.1905 + 1G>A (rs3918290) DPYD*2A c. 2846A>T (rs67376798) *D949V* c.1679T>G (rs55886062) DYPD*13 c.1236G>A (rs56038477) *HapB3*	
Siponimod	*CYP2C9*	c.1075A>C (rs1057910) CYP2C9*3 c.430C>T (rs1799853) CYP2C9*2	Currently not on the National Genomic Test Directory and testing is provided by the manufacturer
Eliglustat	*CYP2D6*	Variants are tested to assign a metabolizer state (poor, intermediate, extensive)	Currently not on the National Genomic Test Directory and testing provided by the manufacturer
Aminoglycosides	*MT‐RNR1*	m.1555A>G (rs267606617) m. 1095T>C (rs267606618) m.1494C>T (rs267606619)	Scottish Genomic Test Directory: only m.1555A>G
Abacavir	*HLA‐A*	HLA‐B*57:01	
Mavacamten	*CYP2C19*	CYP2C19*2 (rs4244285) CYP2C19*3 (rs4986893) CYP2C19*17 (rs12248560)	Not currently in the Scottish Test Directory
Sulphonylurea	*KCNJ11 ABCC8*	Panel testing for genetic diagnosis of neonatal diabetes	Variants in these genes allow transfer from insulin to sulphonylurea in neonatal diabetes
Sulphonylurea and glucagon‐like peptide 1 agonist	*HNF1A* *HNF4A* *HNF1B* *GCK*	Whole exome testing or panel testing for genetic diagnosis of monogenic diabetes	Specified genes predict response to treatment in Maturity Onset Diabetes in Youth (MODY)
Clopidogrel	*CYP2C19*	*2 and *3 loss of function variants	Testing is currently available to select stroke centres as part of an initial pilot phase
Thiopurines	*TPMT* *NUDT15*	c.238G>C (TPMT*2) (rs1800462) c.460G>A (rs1800460) c.719A>G (TPMT*3A) (rs1142345) c.415C>T (NUDT15*3) (rs1166855232)	Testing is available for patients with a confirmed diagnosis of acute lymphoblastic leukaemia and proposed treatment involving purine analogue based drugs

*Note*: Gene–drug pairs where pharmacogenetic testing is currently available within the NHS England testing ecosystem (accurate as of January 2025). Testing availability may be different in Scotland. HLA testing is not available via the NHS England test directory, but is accessible through regional HLA laboratories which are established to support HLA typing for transplantation. Of note, a phenotypic test measuring activity levels of the enzyme thiopurine methyltransferase (TPMT) is mostly used to guide treatment with thiopurines, although genetic testing is also available.

The Personalised Prescribing report recommended that the NHS should transition towards a pre‐emptive panel‐based genotyping approach, with data made available to primary, secondary and tertiary care services.^5^ In 2023 the PGx‐NoE launched the Pharmacogenetics Roll Out: Gauging Response to Service (PROGRESS) Programme (ISRCTN 15390784) which aims to develop, validate and pilot a panel‐based pharmacogenetic service for the NHS in England. By January 2024, the study had recruited ~500 adults and was open for recruitment at 16 GP practices around the country. This project provides a technical and methodological framework for the development of a panel‐based pharmacogenomic programme in the NHS.

The NHS offers whole‐genome analysis primarily to patients with suspected genetic disorder, rare disease, difficult‐to‐treat cancer or children with cancer. The NHS Long Term Plan set a target to sequence 500 000 whole genomes from patients as part of their routine care by 2023/24.^13^ The pharmacogenomic information contained within these data could be extracted for patient benefit in the future.^5^ Similarly, pharmacogenomic information acquired through research avenues such as Our Future Health Project, could be curated and added into patient electronic records for use in different settings and time points during their healthcare journey, although details on how such information will be returned to participants has not been published.

### 2.2 Transition towards reusing pharmacogenetic data over time

To be of value in clinical practice, the results of pharmacogenetic testing must be integrated into a model of service delivery. Broadly, there are three main approaches that have been defined (Table 2).^14^ The first, and most traditional, is reactive, where testing is undertaken in the context of a specific prescription. For example, take a stroke clinic where a patient is due to be prescribed clopidogrel. NICE recommends *CYP2C19* genotype testing to guide clopidogrel use in people who have had recent ischaemic stroke or transient ischaemic attack (TIA).^15^ Using a reactive model of service delivery, the patient might provide a blood sample for *CYP2C19* genotyping, with that result then used to inform antiplatelet prescription once the result is available.

The second approach is reactive with planned reuse (RWPR), where testing is still triggered based on the prescription of a certain medicine, but the results from the pharmacogenetic test are subsequently retained, stored and structured in such a way that can be reused to guide future prescribing activity. Using the same example, a patient presenting to a stroke clinic requiring clopidogrel might have *CYP2C19* testing performed, but this might be part of a broader pharmacogenetic panel. This data can be stored in the patient's health record and subsequently accessed to guide selection of other drugs at some stage in the future.

Finally, pre‐emptive testing represents a further iteration, where a pharmacogenetic test is performed but undertaken outside of a specific prescribing context. This means, in theory, that pharmacogenetic results will be available to help guide future prescribing activity at the point of need, removing the requirement to wait for the laboratory test. In this model, the same patient might present to their stroke clinic, but instead of needing to order a *CYP2C19* test, the data are already embedded within their electronic health record, having been generated previously. This means their prescription can be optimized at their first appointment.

Hypothetically, each of the two testing approaches could be combined with any of the three service models. However, in reality, single gene testing is most commonly embedded within a reactive model of service delivery, whilst panel‐based testing has typically been explored in the context of RWPR or pre‐emptive models of service delivery. At times in the pharmacogenetic literature, the “test” and the “model of service delivery” are conflated, leading to some confusing and contradictory terminology.^8^

### 2.3 Relevant gene–drug associations

Over the past decade, the Clinical Pharmacogenetics Implementation Consortium (CPIC) and a European counterpart, the Dutch Pharmacogenetic Working Group (DPWG), have developed structured methodologies to iteratively review the pharmacogenetic literature and produce standardized clinical guidelines. These documents appraise the available evidence to provide guidance on variant nomenclature, metabolizer classification, testing approaches and, critically, guidelines on dosing and drug choice (for individuals with known genotype). At the time of writing, CPIC have produced 27 guidelines for gene–drug class pairs. These guidelines cover frequently prescribed medicines including antibiotics, antiplatelets, opioids, non‐steroidal anti‐inflammatory drugs (NSAIDs), proton pump inhibitors (PPIs) and anti‐depressants.

The CPIC and DWPG guidelines summarize, based on expert review of the literature, how prescribing practice should be modified in the presence of clinically relevant genetic variation. What they explicitly do not say is when pharmacogenetic testing should take place and what the model of service delivery should be. For example, the guidelines for *RNR1* and aminoglycoside antibiotics state that, in the presence of clinically relevant *RNR1* variation, aminoglycoside antibiotics should be avoided unless the high risk of permanent hearing loss is outweighed by the severity of infection and lack of safe or effective alternative therapies.^16^ The guideline does not state if, when and how testing should take place.

This same approach is consistent throughout international pharmacogenetic guidelines, reflecting an awareness that models of service delivery for pharmacogenetic testing will differ between countries and institutions depending on testing capacity, digital infrastructure and funding availability. Overly prescriptive guidelines stating how and when testing should be undertaken would be of little value in a system where pharmacogenetics is still in its infancy. As such, the ambition of organizations such as CPIC and the DPWG is that individual institutes, or broader healthcare systems, can integrate pharmacogenetic guidelines into their infrastructures as they develop.

The Personalised Prescribing report recommended that there should be centralized selection of key gene–drug pairs for any national rollout in the NHS, with agreed UK guidance and clear eligibility criteria. This is set to be developed through the recently announced Centre for Excellence in Regulatory Science and Innovation in Pharmacogenomics (CERSI‐PGx). This initiative does not aim to replicate guidelines produced by CPIC or the DPWG but rather to tailor these for the UK healthcare and regulatory systems.

### 2.4 Capacity and turnaround time

Approximately 100 000 patients have a new ischaemic stroke or TIA each year and there is good evidence to support genotype guided antiplatelet therapy in neurovascular disease, with alternative agents such as modified‐release dipyridamole and aspirin, or increasingly ticagrelor, used instead.^17^ Tayside, a region in Scotland, was the first area of the UK to introduce routine clopidogrel genotyping for patients with a new stroke or TIA.^18^ This same approach is set to be recommended across the UK by NICE, with a mixed model of laboratory testing and point‐of‐care (PoC) strategies endorsed as a means by which to undertake testing.

If there was complete compliance with NICE guidance, and laboratory testing was offered to all 100 000 patients, this would represent just under 8500 new tests per month and an over 30% increase in NHS Genomic Laboratory Hub workload, as measured by the number of tests reported.^19^ This hints at the potential scale of pharmacogenetic testing, demonstrating how fundamentally different it is from other areas of genomic medicine. Without proper planning and appropriate resourcing, this testing could place excessive demands on an already stretched laboratory service. NICE's assessment affirms the evidence underpinning pharmacogenetics and its potential usefulness as an intervention in the NHS, but, once again, strategies are required to embed this intervention within a model of service delivery so it can be accessed and used across the health system.

Not only is testing demand likely to be significantly higher, but results need to be made available to healthcare professionals in a clinically relevant timeframe. Rare disease testing may take several months, but in many clinical scenarios such a turnaround time would be unacceptable. Following a stroke, a patient should be on their definitive antiplatelet therapy within days to weeks. As such, the genetic data is required within that timeframe. This will require extensive optimization of existing laboratory pathways, or the adoption of near‐patient tools such as rapid PoC devices.^10^

The potential throughput and speed required to operationalize pharmacogenomic testing are just two examples of how, in many ways, pharmacogenomic testing is fundamentally different from how rare disease and cancer services operate. The Personalised Prescribing report recommended that expected testing volumes should be defined not to overload the nascent system, and that capacity should be developed iteratively. Reflecting this, in relation to *CYP2C19* testing, the NoE have now launched a pilot to implement and assess laboratory and PoC testing approaches, beginning at four sites which will inform decisions on wider commissioning in 2025. However, it is highly likely that dedicated pharmacogenomic laboratories will be required to achieve broad and equitable implementation.

## 3 THE DATA

### 3.1 Complexity of interpretation

The structure of a genetic report produced by a genomic laboratory hub is informed by the American College of Medical Genetics (ACMG) standards and guidelines for the interpretation of sequence variants.^20^ This is also reiterated in the UK by the Association for Clinical Genomic Science (ACGS) through their best practice guidelines for variant classification.^21^ These standards provide a reporting framework which will typically include details on the test performed, the approach to interpretation, the sequence variants, the assigned pathogenicity, and any clinical recommendations. Outside of clinical genetics and certain subspecialities, very few healthcare professionals are routinely exposed to these types of reports in their day‐to‐day practice and, despite the best efforts of mainstreaming programmes, very few will have any formal training in the interpretation of genomic variation.

In the context of rare disease and cancer diagnostics, the fact that most clinicians have relatively little awareness of the nuances of genetic test reporting is of limited concern. General practitioners (GPs) have very little need to review and action a rare disease result, and it is not part of an acute physician's role to interpret a somatic cancer report. However, if pharmacogenetic testing is to happen at scale, it will not be in clinical genetics centres that the data are reviewed and used. Rather, it will be in environments like acute medical units and general practice surgeries, where there is neither the time nor expertise to interpret traditional genetic reports.

Stakeholder engagement with both patients and primary care physicians, undertaken as part of the PGx‐NoE, has consistently highlighted that there is a preference for pharmacogenetic data to be presented simply and in the context of a prescription.^22^, ^23^, ^24^ Specifically, the predicted uptake of a pharmacogenetic testing service decreases significantly if the results presented back to the clinician are too broad and not presented as actionable guidance. This has informed the design of the PROGRESS study (ISRCTN15390784), with the development of a proof‐of‐concept information solution which is able to return actionable guidance directly into the patient's electronic healthcare record.

### 3.2 Lack of decision support systems

If pharmacogenetic data are to be used in routine practice, and reused over a patient's lifetime, it is unlikely to be feasible for healthcare professionals to review a traditional genetic test report at each prescribing encounter. To do so would be asking healthcare professionals to (1) be cognisant that their patient has had pharmacogenetic testing, (2) know how and where to access the results in their electronic health records (EHR) (if they are stored there at all), (3) interpret the pharmacogenetic variation in the context of the most up‐to‐date guidance from groups such as CPIC or the DPWG, and (4) use that information to select the most appropriate medicine. This would represent an enormous cognitive and time burden for an already stretched healthcare system. As highlighted in Section 3.1, work undertaken in the UK has shown that such a legacy reporting model would result in extremely poor uptake amongst healthcare professionals.^23^, ^24^ As such, if the ambition is to develop a scalable pharmacogenetic service that has good uptake amongst stakeholders, a new model for interpretation and reporting of pharmacogenetic data is required.

The Personalised Prescribing report recommended that there should be an effort to design interconnected systems that enable access to pharmacogenomic data across the healthcare system. Making pharmacogenetic guidance available at the point of prescribing, within the patient's EHR, should be an ambition of any prospective pharmacogenomic service. However, the rationale for this integration is not because EHRs represent an ideal repository to store and interact with genomic data – in fact, many post‐implementation assessments of EHR systems show huge dissatisfaction with these electronic systems in comparison with their analogue predecessors.^25^, ^26^ A recent assessment of the implementation of the Epic EHR system in Denmark and Finland produced a withering assessment of their experience. It was estimated that more time was spent on common tasks such as finding, ordering tests, finding results, and writing patient notes. This assessment concluded that the “dissatisfying post‐implementation experiences with Epic have replaced grand pre‐implementation expectations.”

A review of the global pharmacogenetic programmes highlight that developing clinical decision support functionality within a patient record is entirely possible. However, it should be noted that the vast majority of implementation efforts have been at single institutions which have their own laboratories and make use of a single EHR provider. This is not the case in the NHS where there is an imperative to share data across institutional boundaries and within different EHR platforms to enable equitable delivery of care. This necessitates an information technology (IT) infrastructure allowing exchange of data between systems, a concept known as interoperability. Amongst other factors, this is reliant on a common, machine‐readable description of clinical data, allowing a semantic understanding between systems exchanging information.^27^, ^28^ To achieve this type of semantic interoperability, clinical concepts need to be converted into technical artefacts that use international standards for health data, such as openEHR, fast healthcare interoperability resources (FHIR) and SNOMED‐CT.^29^ At present, these formalized standards do not exist for pharmacogenetic data, though organizations like the Global Alliance for Genomics & Health (GA4GH) have launched programmes to develop these.

To realize the benefit of pharmacogenomic‐guided prescribing at scale, whilst also ensuring the fidelity of the data and standardization of the guidance, the NHS cannot rely solely on single organization solutions which provide both data management and clinical workflow support as one product (i.e., Epic or Oracle Cerner). The traditional approach to integrating pharmacogenetic data within these systems is to upload the genomic data directly into those platforms, allowing clinical decision support functionality to be built locally. Again, this might be a reasonable solution at a single centre, but in the NHS, it would require the upload of genomic data into a large number of different EHR platforms and the construction of distinct clinical decision support tools as part of each integration. Over time, without careful curation, the guidance issued by these systems would begin to diverge.

Rather than siloing data within EHR systems the NoE and NHS England, within the PROGRESS study, are piloting an approach to store and structure results in separate and accessible informatic repositories. This facilitates a “vendor‐neutral” data platform approach based on standards, allowing implementation of pharmacogenetics by providing access to pharmacogenetic data as and when required through application programming interfaces (APIs). Providing the different EHR and clinical decision support providers are willing to collaborate with the NHS to develop this integration, this approach would mean there was standardization of the interpretation of variants and of the guidance itself. This could lay the foundations for an innovative ecosystem where health IT suppliers compete on features and functionalities, rather than isolating health data. Providing incentives for large multinational informatic organizations to engage will require organizations such as NHS England to develop innovative commissioning and reimbursement models, reflecting the novelty of this approach.

## 4 DESIGNING A CLINICAL SERVICE

### 4.1 Conflicting evidence for clinical effectiveness

There is robust evidence in the literature, curated by groups like CPIC and DPWG, for the implementation of pharmacogenetic‐guided prescribing for a number of single gene–drug pairs. However, for panel‐based pharmacogenetic testing embedded in a model of service delivery which facilitates data reuse, clinical outcomes can be challenging to assess beyond the index prescription which precipitated the test. The PREPARE trial recently undertook an assessment of panel‐based pharmacogenetic testing across Europe.^8^ Even this large trial, however, focused only on adverse drug reactions (ADRs), and did not capture impact on effectiveness, following up patients for a maximum of 18 months. As such, uncertainties remain regarding the impact of implementing panel‐based pharmacogenetics over longer periods of time in the NHS.

It should be noted that there was critique of the PREPARE manuscript for aspects of its statistical analysis, with some correspondents noting that in the genotype‐guided arm a decrease in ADRs was also observed in those patients who did not carry actionable variants.^30^, ^31^, ^32^ The authors argued that this implied that the effect seen in PREPARE was not actually related to genotype‐guided prescribing, but rather to other behavioural or psychological variables associated with someone undergoing genotype‐guided prescribing. The PREPARE authors issued a rebuttal, highlighting the statistical complexities introduced by the real‐world design and showed that the reduction in ADRs for those without actionable variants disappeared when you control for the case‐mix.^33^ Clearly for any implementation programme, be it a single institution or across an entire healthcare system, evidence on the impact of pharmacogenetic‐guided prescribing should continue to be generated in the context of different clinical contexts, the impact of pharmacogenetics on the effectiveness of medication and clinical outcomes and leveraging real‐world data from initiatives like the NHS secure data environments. Developing a learning health system would then allow for further feedback to continually refine the implementation programme, and thereby patient outcomes in a timely and cost‐efficient manner.

### 4.2 Uncertainty regarding cost effectiveness

There is increasing evidence from cost effectiveness analysis (CEA) that pharmacogenetic testing may be cost effective in some jurisdictions and applications.^34^, ^35^, ^36^ However, at the time of writing, no CEA has been undertaken to assess the impact on costs to the NHS, and outcomes for patients, of implementing pharmacogenetic panel testing in primary care. However, an assessment of a multigene human leukocyte antigen (HLA) panel to prevent serious adverse drug reactions in a UK healthcare context showed that panel testing was both cost‐saving and more effective than standard care.^37^

Undertaking a CEA for single gene–drug interactions is a relatively addressable challenge, with the intervention and outcome measures readily definable. Many such studies have been undertaken, with many providing replicable evidence of cost‐effectiveness. As part of the NICE assessment for *CYP2C19* genotype testing to guide clopidogrel use after ischaemic stroke or TIA (DG59), health economists found that pharmacogenetic testing (the intervention) dominated no testing in probabilistic analysis (it cost less and improved patient outcomes), driven by a reduction in secondary vascular events (the outcome).

As clinical implementation moves towards panel‐based testing and service models which reuse data over time, modelling the impact of this intervention becomes increasingly complex. What makes pharmacogenetics somewhat distinct in comparison to other genetic interventions is how a single test can, in theory, be reused throughout a patient's life and in multiple different clinical contexts. The ability to reuse this data over time to guide future prescribing practice is a key attribute favoured by public and clinical stakeholders and could unlock major benefits for the health system.^22^ However, as highlighted in Section 4.1, capturing these longitudinal outcomes is complex and efforts should be made to access routinely collected healthcare data to support this analysis. Without these, undertaking a detailed CEA is challenging. Furthermore, failing to capture prescribing practice outside of a single clinical episode likely produces an underestimate of clinical utility and does not reflect the model of service delivery preferred by stakeholders.

### 4.3 Lack of public and healthcare professional awareness

In the NHS, there is considerable interest at a policy level around using genomics in routine healthcare to predict risk and guide decision making, building on the significant expertise which exists in the UK in relation to rare disease and cancer services. This is often referred to as “mainstreaming”, a term first used in 2003 by the then Secretary of State for Health, John Reid, when discussing the “Our Inheritance, Our Future” white paper.^38^ Published just a few years after the completion of the Human Genome Project (HGP) and buoyed by its success, the report argued that, to achieve its full potential, genomics would need to move from the narrow confines of clinical genetics into clinics around the country. Interestingly, the report highlighted pharmacogenetics as the area likely to have “greatest impact” in the “shorter term”. The report recommended that using genetics in routine care would require the education and upskilling of a wide range of non‐specialist health professionals. In the two decades since the publication of this report, a large number of initiatives, strategies and organizations have been created to educate the NHS workforce about genetic medicine.^39^, ^40^, ^41^

The Genomics Education Programme, representing the present vehicle for delivering genetics education in the NHS, defines mainstreaming as “pre‐test counselling and consent processes being undertaken at the point of care by non‐geneticists”. This is a prevailing view, reflecting the fact that the genetic mainstreaming agenda and its associated strategies are almost exclusively viewed through the lens of rare disease and cancer.^42^ Mainstreaming is often reduced to non‐geneticists doing things that geneticists normally do; counselling, ordering tests, interpreting reports, giving results, and cascade screening. This is commendable and may take some burden away from stretched clinical genetics services, but this interpretation of mainstreaming is limited.

In many ways the use of pharmacogenetics in routine practice represents a new healthcare paradigm, fundamentally distinct from the traditional genetics consultation which has historically served as the model for mainstreaming. These can be summarized as two key differences: the potential scale of pharmacogenetics as an intervention, impacting testing and consenting models, and the clinical context in which the data is used, impacting reporting and interpretation models. As such, any educational resources should be proportionate and developed to support service users. One example of a potentially powerful tool are “just‐in‐time” learning resources, such as those developed within the Genomics Education Programme's GeNotes initiative which now has approximately 30 pharmacogenomic resources.^43^ Within PROGRESS, these have been integrated into clinical decision support notifications, meaning these resources are surfaced at the point of need. There is likely to be a need for clinical champions with relatively deep expertise in pharmacogenomics, and clinical pharmacologists and pharmacists are well placed to undertake such a role. However, for the vast majority of healthcare professionals, pharmacogenomics needs to be integrated seamlessly with their day‐to‐day practice and made simple, otherwise uptake will be severely impeded.

## 5 CONCLUSION

Technological advances and an increasing understanding of genomic variation have long been predicted to herald a healthcare revolution. In reality, the promise outlined at the completion of the Human Genome Project, that the “diagnosis, prevention and treatment of most, if not all, human diseases” will be revolutionized by genomics has not been fully realized. In part, this is because no matter how advanced a genetic laboratory technology might be, it must be incorporated and contextualized within end‐to‐end healthcare systems, with all their nuances, imperfections and inefficiencies.

The theoretical clinical utility of pharmacogenomics has been demonstrated across multiple studies, and the longitudinal relevance of an individual's genomic data throughout their lifetime has been well established. There appears to be broad acceptability for pharmacogenomic testing from both public and clinical stakeholders, but how any service is designed has a meaningful impact on uptake. Any implementation effort at scale in the UK will require multidisciplinary input, bringing together clinicians, academics and industry partners. Health services must also ensure that services meet the requirements and expectations of the diverse populations they often serve. This consideration should run across the end‐to‐end service, from the laboratory tests, which should be designed to assess relevant variants found in different biogeographical groups, to commissioning which should promote equality of access.

These joint workshops between the PGx‐NoE, the UK Pharmacogenetics and Stratified Medicine Network and the BPS highlight the significant progress that has been made over the last 5 years. There remain considerable barriers towards routine, evidence‐based, implementation of pharmacogenomics, but there are increasingly tangible pathways to make this a reality. The UK has world‐class genomic testing capabilities and has previously demonstrated its commitment to innovation in healthcare, having developed and successfully delivered the 100 000 genomes project. There is an opportunity for the NHS to become a global leader in genomics‐informed healthcare, and the potential rewards are considerable. Effective implementation would shift the focus from sickness to health, improving outcomes for patients and reducing burden on the healthcare system.

### 5.1 Nomenclature of targets and ligands

Key protein targets and ligands in this article are hyperlinked to corresponding entries in http://www.guidetopharmacology.org and are permanently archived in the Concise Guide to PHARMACOLOGY 2021/22.^44^

## AUTHOR CONTRIBUTIONS

J.H.M. and M.T. drafted the manuscript with critical review from W.G.N. and P.M. All authors approved submission of the final manuscript.

## CONFLICT OF INTEREST STATEMENT

M.P. currently receives partnership funding, paid to the University of Liverpool, for the following: MRC Clinical Pharmacology Training Scheme (co‐funded by MRC and Roche, UCB, Eli Lilly and Novartis), and the MRC Medicines Development Fellowship Scheme (co‐funded by MRC and GSK, AZ, Optum and Hammersmith Medicines Research). He has developed an HLA genotyping panel with MC Diagnostics but does not benefit financially from this. He is part of the IMI Consortium ARDAT (www.ardat.org); none of these of funding sources have been used for the current research. WN and JM are co‐founders of an early‐stage health technology start‐up, Fava Health Ltd.

## Data Availability

Recordings of the Network of Excellence meetings which informed this manuscript can be found on the North West Genomic Medicine Service Alliance Website (https://www.nw-gmsa.nhs.uk/media-news-and-events/events/national-pharmacogenomic-network-excellence-events).
